# AttentionCARE: replicability of a BCI for the clinical application of augmented reality-guided EEG-based attention modification for adolescents at high risk for depression

**DOI:** 10.3389/fnhum.2024.1360218

**Published:** 2024-07-09

**Authors:** Richard Gall, Nastasia Mcdonald, Xiaofei Huang, Anna Wears, Rebecca B. Price, Sarah Ostadabbas, Murat Akcakaya, Mary L. Woody

**Affiliations:** ^1^Signal Processing and Statistical Learning Laboratory, Department of Electrical and Computer Engineering, University of Pittsburgh, Pittsburgh, PA, United States; ^2^Clinical Application of Neuroscience Laboratory, Department of Psychiatry, University of Pittsburgh, Pittsburgh, PA, United States; ^3^Augmented Cognition Laboratory, Department of Electrical and Computer Engineering, Northeastern University, Boston, MA, United States

**Keywords:** affect-biased attention, electroencephalography (EEG), augmented reality (AR), brain-computer interface (BCI), neurofeedback, depression

## Abstract

Affect-biased attention is the phenomenon of prioritizing attention to emotionally salient stimuli and away from goal-directed stimuli. It is thought that affect-biased attention to emotional stimuli is a driving factor in the development of depression. This effect has been well-studied in adults, but research shows that this is also true during adolescence, when the severity of depressive symptoms are correlated with the magnitude of affect-biased attention to negative emotional stimuli. Prior studies have shown that trainings to modify affect-biased attention may ameliorate depression in adults, but this research has also been stymied by concerns about reliability and replicability. This study describes a clinical application of augmented reality-guided EEG-based attention modification (“AttentionCARE”) for adolescents who are at highest risk for future depressive disorders (i.e., daughters of depressed mothers). Our results (*n* = 10) indicated that the AttentionCARE protocol can reliably and accurately provide neurofeedback about adolescent attention to negative emotional distractors that detract from attention to a primary task. Through several within and cross-study replications, our work addresses concerns about the lack of reliability and reproducibility in brain-computer interface applications, offering insights for future interventions to modify affect-biased attention in high-risk adolescents.

## 1 Introduction

Attention plays a critical role in shaping an individual's ongoing perceptions about their environment and their ability to distinguish between cues related to sadness vs. joy or punishment vs. reward (Posner, 2016). This, in turn, has profound downstream effects on behavioral patterns and emotional states (Beck, 2008). Affect-biased attention refers to a biased pattern of preferential attention toward a stimulus based on its relative affective salience (Todd et al., 2012; Morales et al., 2016). This cognitive phenomenon is driven by biobehavioral mechanisms that prioritize stimulus-driven attention to emotionally salient stimuli, which diverts goal-directed attention from stimuli relevant to a task at hand (for a review, see Woody and Price, 2022). For example, an individual who exhibits high levels of stimulus-driven attention for negative information may be more likely to click on a text notification about a distressing news article while completing an academic assignment, detracting from goal-directed attention. When this bias happens chronically, it can lead to an increased risk for depression or other internalizing disorders (LeMoult and Gotlib, 2019).

Affect-biased attention for negative information is theorized to be a key contributor to the development and maintenance of depression and anxiety. According to cognitive models of depression, increased attention toward negative information leads to a skewed integration of environmental information across cognitive systems (Price and Woody, 2024). This imbalance gives rise to a flow of negative memories and perceptions regarding oneself, others, and the world, which then increases the likelihood that the individual will continue to exhibit biased attention for negative information. Empirically, cognitive models have been supported by research showing that increased attention to negative information—particularly when it occurs at the expense of positive and/or goal directed information—are associated with depressed mood and functional impairment (for meta-analyses, see Peckham et al., 2010; Armstrong and Olatunji, 2012; Suslow et al., 2020).

Although much of past research has centered on adults, a growing body of evidence suggests a comparable link between affect-biased attention and adolescent depression (Platt et al., 2017). For example, studies have shown that adolescents with a history of past or current depression, when compared to their never-depressed peers, are more likely to display increased attention to sad stimuli (Ladouceur et al., 2006; Hankin et al., 2010; Maalouf et al., 2012; Sylvester et al., 2015). Further, the severity of adolescent depressive symptoms is correlated with the magnitude of such affect-biased attention (Platt et al., 2015; Sylvester et al., 2015). Finally, affect-biased attention for negative information has been shown to be a marker of risk for future adolescent depression, as increased attention for sad stimuli is already present in never-depressed but high-risk adolescents, but not in their lower risk peers (Joormann et al., 2007), and this bias can be used to predict future trajectories of adolescent depressive symptoms (Osinsky et al., 2012).

Affect-biased attention often first emerges during adolescence (Gibb et al., 2023) and is thought to have an outsized impact on adolescent mood for several reasons. First, adolescence represents a critical phase for brain maturation. During this period, the development of prefrontal cortex regions, which modulate goal-directed attention, is ongoing (Ladouceur, 2012; Casey et al., 2019). Simultaneously, limbic regions, which prioritize processing of affectively salient stimuli, exhibit heightened activity during adolescence, compared to childhood or adulthood. This heightened reactivity can often overwhelm still maturing “goal-directed” regulatory inputs from the prefrontal cortex and fronto-limbic connections responsible for sustaining task-oriented attention, leading to affect-biased attention (Ladouceur, 2012; Casey et al., 2019; Woody and Price, 2022). Second, adolescence is defined by a series of changes such as the onset of puberty, social transitions, and increasing academic demands (Silk et al., 2012; Guyer, 2020) that can lead to novel stressors that are no longer well-addressed by the coping skills established during childhood (Rapee et al., 2019). Thus, during adolescence, the confluence of heightened stress and ongoing brain maturation can allow affect-biased attention for negative information to flourish, which may then increase risk for future depression (LeMoult and Gotlib, 2019).

Identifying markers of risk for adolescent depression is seen as a critical goal for public health as these markers may also inform novel intervention targets that could alter the trajectory of depressive disorders (Woody and Price, 2022). Adolescence marks a key developmental window of risk for the onset of depressive disorders given that levels of depression rise precipitously during this phase and as many as 17% of adolescents will experience an episode of major depressive disorder (MDD) by adulthood (Merikangas et al., 2009). Furthermore, research has indicated that the likelihood of adolescent depression is increased in specific populations. For example, adolescent girls are more than twice as likely to develop MDD compared to boys (Rapee et al., 2019). Additionally, offspring of depressed mothers are 3–4 times more likely to develop MDD than those with never-depressed mothers (Goodman, 2007). Given the elevated risk for depression during adolescence, especially among high-risk youth like girls and offspring of depressed mothers, it is imperative to identify risk factors that precede the onset of adolescent depression, as they may inform modifiable targets for early preventative interventions that mitigate the risk of depression.

For these reasons, our team sought to develop a novel brain-computer interface (BCI), AttentionCARE, that could be used to modify affect-biased attention for negative information in a population of high-risk adolescents (i.e., adolescent daughters of depressed mothers). We hypothesized that training high-risk adolescents to redirect attention from negative information and attend to goal-directed information might disrupt the vicious cycle linking affect-biased attention and depressed mood. Initial interest in modifying affect-biased attention to improve depressed mood stemmed from observations that antidepressants, such as selective serotonin reuptake inhibitors (SSRIs), led to reductions in attention to negative information, preceding improvements in mood (Browning et al., 2010). Attention bias modification training (ABMT) has since been tested as a possible treatment for depression, and a recent meta-analysis from adult samples revealed that ABMT leads to significant reductions in depressive symptoms (Xia et al., 2023). However, this review and others (Price et al., 2015; Rodebaugh et al., 2016) have also shown that existing ABMT paradigms are limited by reliability and interpretability challenges, and there is a need to enhance the robustness and precision of these interventions. Further, ABMTs have rarely been tested during adolescence (but see De Voogd et al., 2014; LeMoult et al., 2016).

Due to the accuracy of brain-based measurements, neurofeedback has emerged as a promising approach for modifying affect-biased attention (Woody and Price, 2022). Past research has demonstrated the effectiveness of using neurofeedback to influence real-time attentional patterns (Debettencourt et al., 2015), which can enable depressed patients to promptly and precisely modulate their affect-biased attention (Schnyer et al., 2015; Mennen et al., 2021). However, these previous neurofeedback paradigms have relied on expensive functional magnetic resonance imaging (fMRI) procedures, constraining their future applicability to clinical settings.

Using electroencephalogram (EEG) based neurofeedback can not only enhance cost-effectiveness but also increase the feasibility of clinical translation. Thus, in a previous pilot study, we developed a more cost-effective EEG brain-computer interface, which used steady-state visual evoked potentials (SSVEPs) to direct the attention of five healthy adults while they completed a primary cognitive task in augmented-reality (AR) (Huang et al., 2022). Derived from EEG, SSVEPs are evoked by visual stimuli that are luminance modulated at a fixed frequency and generated by the primary visual cortex (Wieser et al., 2016). The magnitude of SSVEP responses fluctuate with an individual's attention to a flickering stimulus, and by frequency-tagging SSVEPs to multiple visual stimuli flickered at distinct frequencies, this method can effectively differentiate attention to competing stimuli, even when they overlap entirely in both time and space (Müller et al., 2008; Woody et al., 2017). SSVEPs offer BCIs a temporally-sensitive neural measure of competition between stimulus-driven and goal-directed attention at the level of neuronal populations in the visual cortex.

Our pilot AttentionCARE BCI integrated SSVEP measurements, captured in response to competing visual stimuli presented through AR technology, for real-time detection of affect-biased attention and implementation of neurofeedback. This BCI employed a Microsoft HoloLens AR head-mounted display to present competing visual stimuli superimposed on the real-world environment. In a clinical context, the use of AR enables patients to perceive their surroundings while tasks or alerts are displayed in their visual field, creating an overlay effect. This approach enhances comfort and intuitive control compared to an entirely virtual space. Notably, AR technology has found applications in medical settings and as part of BCI protocols (Lenhardt and Ritter, 2010; Zao et al., 2016; Mak et al., 2022). Despite these advancements, our pilot BCI was the first to employ AR as a part of affect-biased attention training (Huang et al., 2022).

To direct affect-biased attention training, we developed an attentional paradigm where, in each trial, we superimposed two competing visual stimuli on the Hololens AR goggles. Participants were instructed to focus on the task-relevant stimulus (a semitransparent group of parallel lines, or a “Gabor” patch) while ignoring an emotional distractor (an angry or sad face). Each stimulus flickered at a separate frequency (8.57 or 12 Hz) to differentiate SSVEP responses to the Gabor and the face. Following each trial, participants received feedback on the extent of attention given to the Gabor vs. the face, as indicated by a calculated SSVEP feedback score. Findings revealed that our AttentionCARE BCI led to discriminant SSVEP responses for both the Gabor and the face and produced excellent internal reliability, when comparing responses to odd vs. even trials (Huang et al., 2022).

The current study sought to implement a similar BCI to target affect-biased attention in a sample of adolescent girls at high risk for depression (*n* = 10). Specifically, we investigated the use of a participant-specific support vector machine (SVM) to generate feedback about the probability that a participant was attending to the Gabor, relative to the emotionally distracting face. Since EEG signals are known to vary across individuals (Schirrmeister et al., 2017; King et al., 2018), a generalized algorithm—such as what was used in our earlier work (Huang et al., 2022) —may not accurately capture the visual attention of all participants. We theorized that by personalizing feedback about affect-biased attention, then it would better reflect the visual attention of a participant and thus better promote learning due to the improved feedback.

Through use of the proposed algorithm, we hypothesized the successful reproduction of our pilot findings showing that discriminant SSVEP responses can be generated by our AttentionCARE BCI with high levels of internal reliability, but now in a sample of adolescents at high risk for future depression (i.e., adolescent daughters of depressed mothers) and with a participant-specific SVM. From a preliminary sample of five adolescents, the performance of several SVMs (using different feature types, kernels, and a forward feature selection technique) were profiled to determine the best performing algorithm across the preliminary group. Following the selection of a novel SVM classifier, we hypothesized that accuracy would be maintained, within a tolerance, by implementing the same SVM classifier parameters in a separate replication sample of five adolescents. If these hypotheses were supported, then the current study's use of a novel BCI, coupled with robust methodology and commitment to reproduce and replicate findings from our past and ongoing work, would ensure a valuable contribution to the development and implementation of a novel BCI to target affect-biased attention in a sample of adolescents at high risk for future depression.

## 2 Methodology

### 2.1 Participants

Participants included 10 adolescents (100% assigned female at birth) who were recruited as part of a larger longitudinal study (*n* = 93). At the time of completing the current protocol, adolescents were between the ages of 14 and 16 (*M* = 14.6). Among adolescents in the current study, 80% self-identified their race as White, 10% as African American/Black, and 10% as Multiracial. Regarding ethnicity, 10% self-identified as Hispanic. The sample was enriched for future risk for depression as 70% of adolescents had a mother with a history of MDD in their lifetime but at the time of completing the protocol, the adolescent did not meet DSM 5 diagnostic criteria for any current or past mood disorder.

Participants were split into two cohorts, consisting of five adolescents in the first cohort and five adolescents in the second. The first cohort was used as the preliminary sample whereas the second cohort was used as a replication sample. The preliminary sample completed only the two baseline phases of the AttentionCARE protocol whereas the replication sample completed both baseline phases and the feedback phase. This study followed an IRB-approved protocol from the University of Pittsburgh.

### 2.2 AttentionCARE protocol

**Figure 1:**
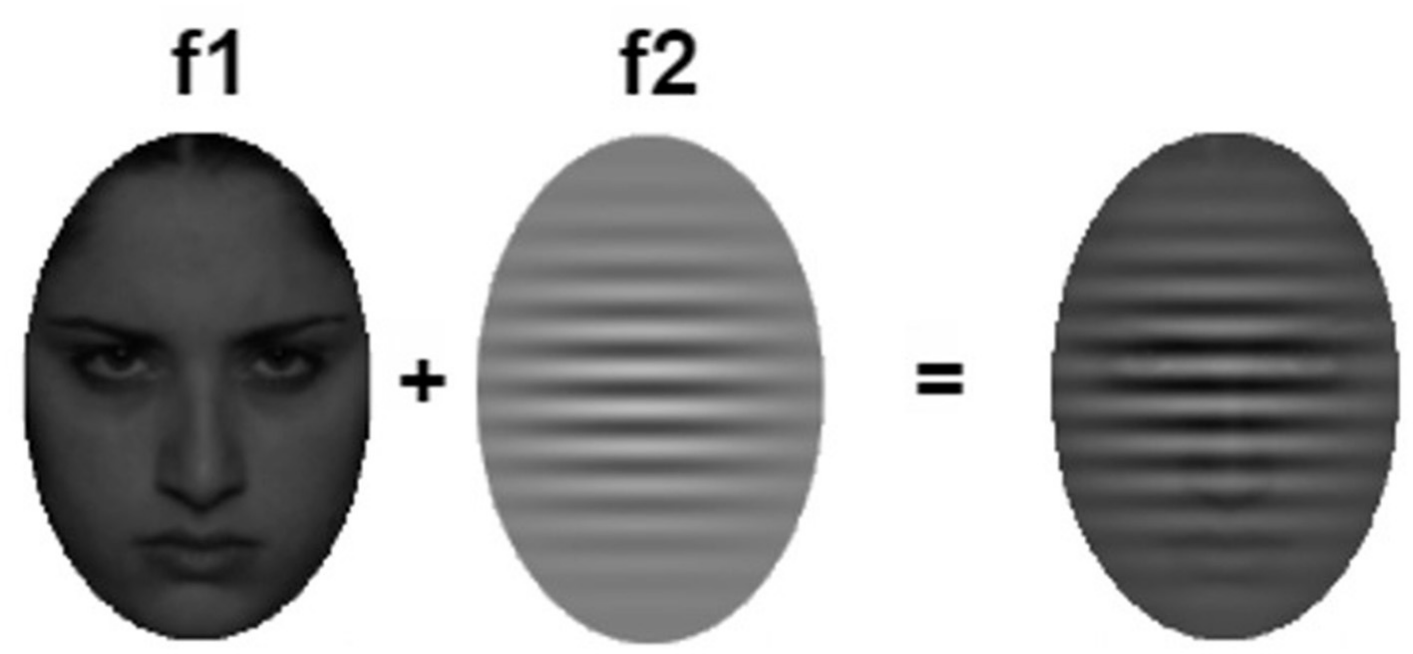
The AttentionCARE stimuli presentation. A Gabor with 50% opacity, flickered at 12 Hz, f_1_, overlays a flickering emotional distractor stimulus at 8.57 Hz, f_2_, (Huang et al., 2022). Facial image reproduced with permission from NIMH Child Emotional Faces Set (Egger et al., 2011).

Consistent with our previous work (Huang et al., 2022), we implemented the EEG-based AttentionCARE protocol using AR, to ascertain participants' attention to affective distractors vs. task-relevant stimuli. In the protocol, each trial consists of an angry or sad face (i.e., affective distractor) presented at the center of the field of view of the AR headset (corresponding to the center of the participants field of view), with a semi-transparent Gabor patch (i.e., task-relevant stimuli) overlaying the face, presented for 5 s. The faces were flickered at a frequency of 8.57 Hz (f_1_) and the Gabor at a frequency of 12 Hz (f_2_). By flickering the stimuli at different frequencies, SSVEPs were evoked in the EEG signal at the specific frequency tag, f_1_ or f_2_, depending on the allocation of the participant's attention to each visual stimulus. Affective distractors included 30 pictures of 15 female adolescent actors with sad and angry expressions selected from the NIMH Child Emotional Faces Set (Egger et al., 2011). An example of the stimuli presented in a trial is depicted in Figure 1.

**Figure 2:**
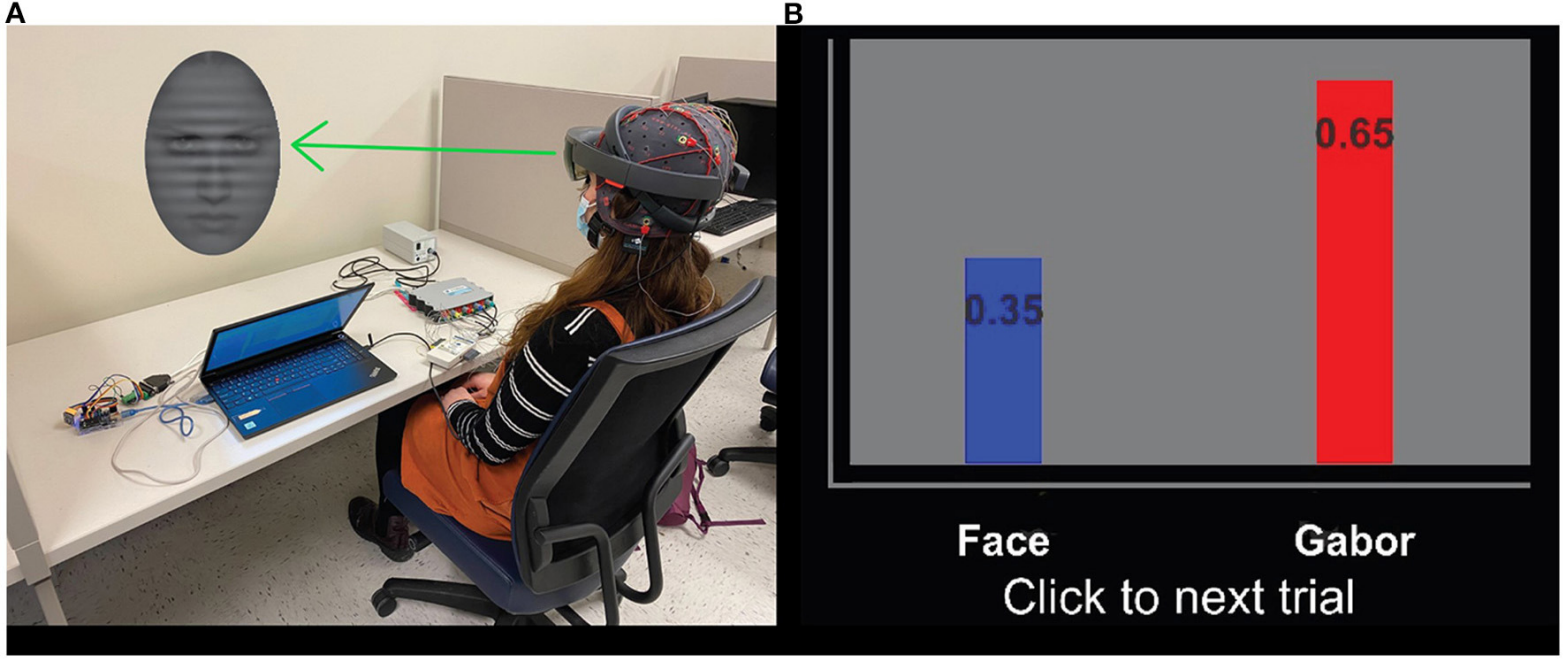
**(A)** Participant completing a trial of the AttentionCARE protocol, depicting an example of the stimuli presented in a trial. **(B)** The bar chart used to display the attention of the participant, where a higher score represents more attention given to a specific stimulus (Gabor or angry/sad face) (Huang et al., 2022).

**Figure 3:**
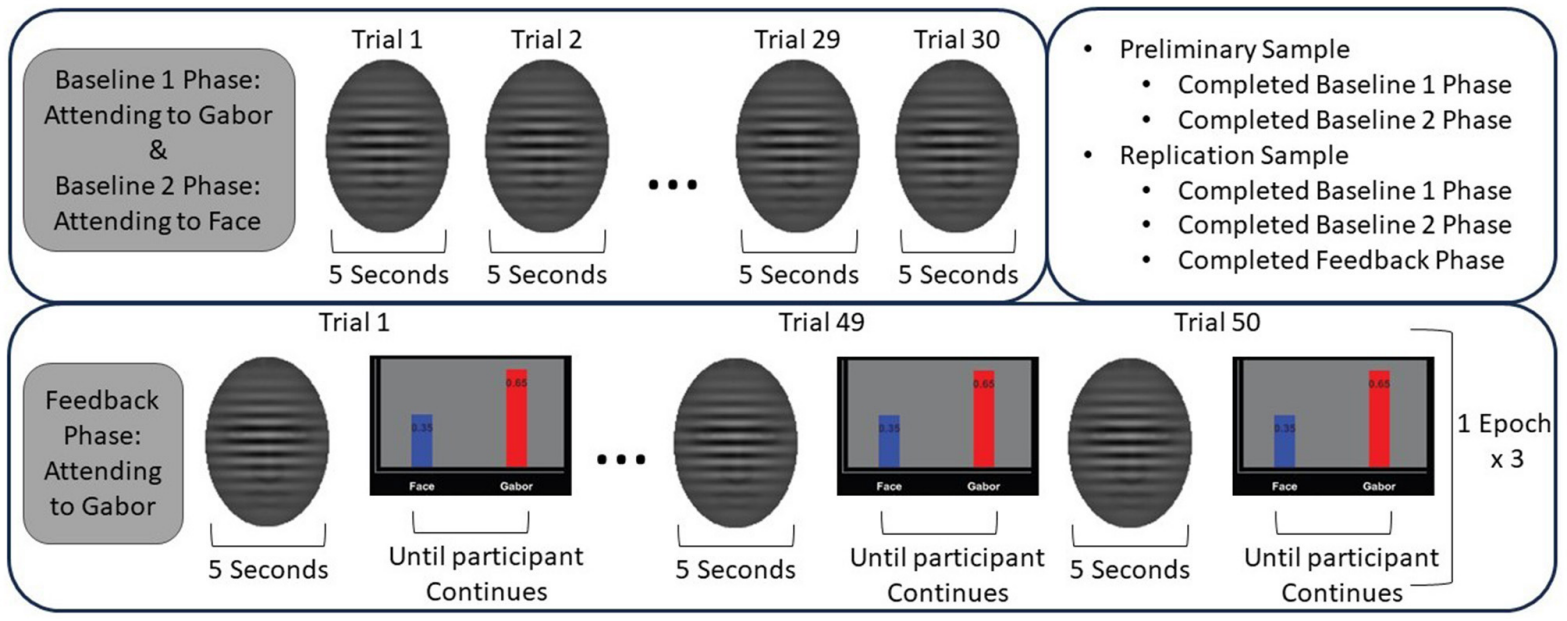
Flow diagram of the AttentionCARE protocol. The facial and feedback stimuli shown here are all the same, but in the protocol the stimuli would vary.

The protocol was comprised of three phases: baseline, feedback, and mastery; however, in this work we focused on the baseline and feedback phases only. The baseline phase consisted of 30 trials, during which the participant was instructed to attend to either the face or the Gabor for every trial. The baseline phase is repeated twice to have the participant attend to both the faces and Gabor for 30 trials each. In “Baseline 1,” participants are asked to pay attention to the Gabor and ignore the face, and in “Baseline 2” they are asked to pay attention to the face and ignore the Gabor. EEG data is collected continuously. During the feedback phase, participants are told to only pay attention to the Gabor and ignore the face during each trial. The feedback phase includes three ‘epochs,' where each epoch consists of 50 trials of stimuli presentation, with feedback presented at the end of each trial. Within each epoch, 10 faces are shown, and each face is shown five trials in a row, which provides an opportunity for the participant to repeatedly practice reducing their attention to that specific face. At the end of the trial, participants are given feedback related to competition between the attention they paid to the Gabor vs. the face. Feedback is given as a visualization of the probability of whether the participant was paying more attention to the face or the Gabor during that trial. Figure 2 depicts an example of feedback given to a participant. The goal of the feedback is to direct participants' attention away from affective distractors (i.e., the faces) and toward the task-relevant stimuli (i.e., the Gabor). Feedback is generated using the EEG data collected during a given trial. A flow diagram of the paradigm is depicted in Figure 3 to better show the different phases of the protocol and where participants were instructed to direct their attention. Additionally, this figure includes the specifics of which cohort completed which phases of the protocol. The display order of the faces is randomly permuted, for each phase, across participants to ensure there is no effect based on the display order.

### 2.3 Acceptability questionnaire

Following the completion of the AttentionCARE protocol, participants completed a self-reported questionnaire to gauge the acceptability of the system [adapted from Sekhon et al. (2022)]. Components of acceptability assessed included: (1) comfort, (2) effort, (3) alignment with values, (4) perceived effectiveness, (5) understanding of intervention, (6) self-efficacy and confidence, (7) opportunity costs. Participants responded to statements, on a scale of 1–5, such as “How comfortable did you feel when you were asked to use neurofeedback to focus your attention on the non-emotional parts of the game (i.e., the group of lines) vs. the negative parts (i.e., the sad and angry faces)?” The anchor point for all questions was 1, representing the minimum for the statement; in the example statement above a response of 1 would represent “very uncomfortable.” Full statements and the *M* (SD) of participant responses are included in Table 1.

**Table 1:** Acceptability Questionnaire Items and *M* (SD) of participant responses.

**Item**	***M* (SD)**
How comfortable did you feel when you were asked to use neurofeedback to focus your attention on the non-emotional parts of the game (i.e., the group of lines) vs. the negative parts (i.e., the sad and angry faces)?	3.60 (1.14)
How much effort did it take to use neurofeedback to focus your attention on non-emotional vs. negative parts of the game?	3.40 (1.34)
How fair is it to ask you to use neurofeedback to focus your attention on non-emotional vs. negative parts of the game?	3.40 (1.34)
It is clear to me how the neurofeedback training will help me to control my attention to non-emotional vs. negative information	2.80 (1.10)
Neurofeedback training has helped me control my attention to non-emotional vs. negative information	3.20 (1.10)
How confident did you feel using neurofeedback to focus your attention on non-emotional vs. negative parts of the game?	2.60 (1.34)
Learning to control my attention to non-emotional vs. negative information is in line with my priorities	2.80 (1.10)

### 2.4 BCI hardware

**Figure 4:**
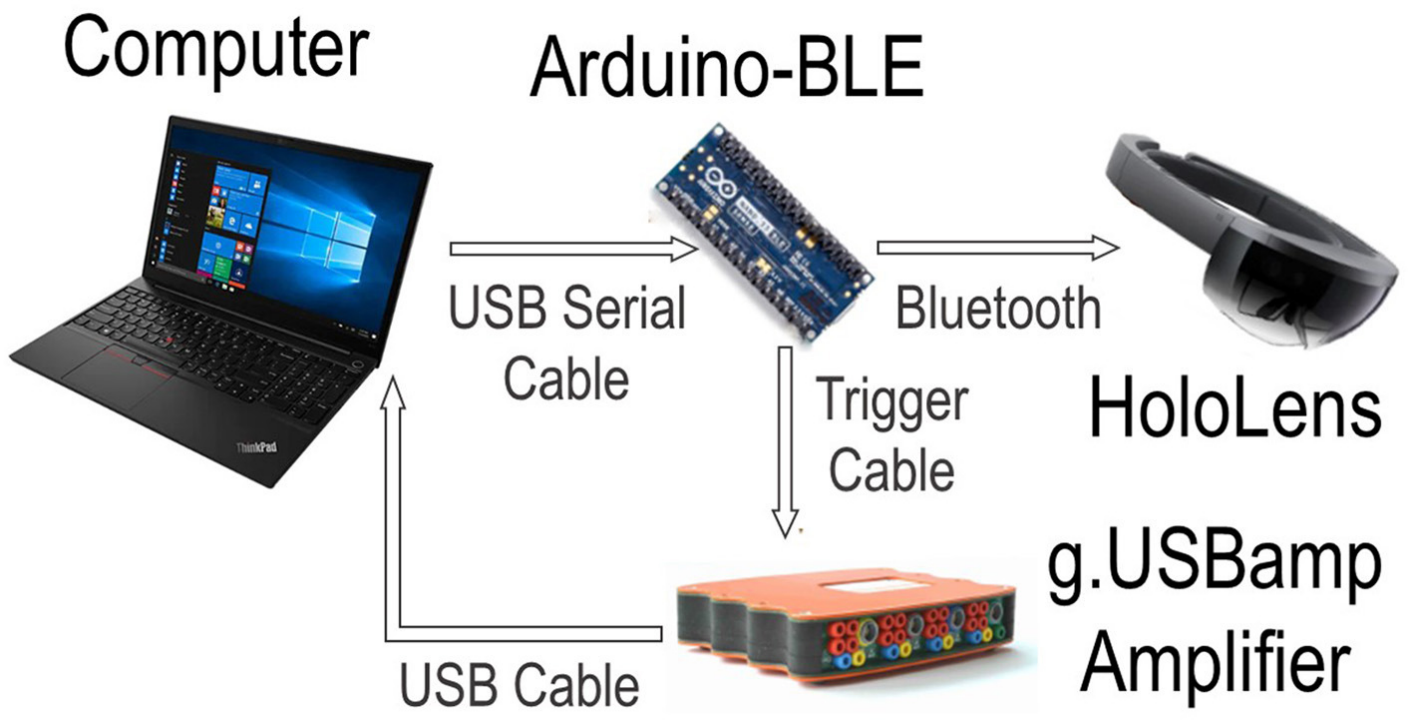
A flowchart illustrating the system's interconnected components. The computer serves as the central control hub for all external devices. The HoloLens handles stimulus presentations, while the G.USBamp Amplifier is utilized for collecting EEG data. The Arduino facilitates communication between the Computer, HoloLens, and Amplifier, ensuring synchronization of stimulus presentation and EEG data collection.

The system is comprised of four components working together to implement the protocol. More specifically a Computer, a Microsoft HoloLens 1, an EEG amplifier, and an Arduino. Figure 4 shows the interconnections of all components. The first component is the computer, which acts as a centralized control hub for all external devices using MATLAB R2015a. The HoloLens uses real-time head tracking to project the stimuli and feedback into a participant's field of view, developed in Unity. The system uses a g.USBamp biosignal amplifier using active g.Butterfly electrodes with cap application from g.Tec (Graz, Austria) to record the EEG signals, at a sampling rate of 256 Hz. The EEG data was collected from 16 channel locations (P1, PZ, P2, CP1, CPZ, CP2, CZ, C3, C4, T7, T8, FC3, FC4, F3, F4 and FZ) based on the international 10/20 system. All EEG data is filtered by a Kaiser FIR bandpass filter between [1, 40] Hz before processing. To connect all components together an Arduino is utilized to send commands via Bluetooth Low Energy (BLE) from the computer to the HoloLens and amplifier. Additionally, due to latency differences between the wired connection of the amplifier and BLE the Arduino synchronizes the EEG data to the stimuli presentation. For a full description of the system refer to our previous work (Huang et al., 2022).

### 2.5 Feedback calculation for AttentionCARE protocol

#### 2.5.1 Preliminary sample

The preliminary sample was used to investigate the initial performance of a participant-specific support vector machine (SVM) to calculate feedback regarding the probability that a participant was attending more to the Gabor or the face based on their SSVEP responses for a given trial. Several different features were extracted, along with a forward feature selection technique, for each participant to establish the optimal features for the model. Three different features were tested: the power spectral density (PSD), the power of EEG signals from different frequency bands around the harmonics of the Gabor and the face, normalized by the total power of the signal (Power banks), and the correlation of the EEG signal to a cosine signal oscillating at the frequencies of the harmonics of the Gabor and the face (Cosine correlation). The features were extracted from the occipital scalp region, which is the region that is responsible for visual perception, corresponding with channels O1, O2, and Oz. The forward feature selection method iteratively adds random features from the full set to an empty feature bank and trains the model until a criterion is met, in this case, the misclassification rate was minimized. This results in a model that uses a minimal subset of features while maintaining a low misclassification rate. In addition to the three different feature types, three different kernels were explored as well: linear, polynomial, and radial basis function (RBF). Due to the limited amount of data (i.e. 60 trials per participant) when training the models, five-fold cross-validation was utilized to not overfit to a certain data split.

Each feature was extracted from the EEG data based on the equations shown in Table 2, where *x*_channel_(*t*) is the EEG data from the respective channel, *X*_channel_(*f*) is the fast Fourier transform (FFT) of the EEG data for a given channel, *N* is the total number of EEG samples, $\hat{f}$ is the fundamental frequency of the Gabor or the face (corresponding to f_1_ or f_2_), *n* is the *n*-th harmonic, and f_0_ is half the range of the frequency bin. For the PSD features, the FFT is taken with respect to the average across the occipital channels discussed above; the power bank and cosine correlation features were extracted from each independent channel (i.e O1, O2, and Oz).

**Table 2:** Equations used to extract features from the EEG data for all different feature types.

**PSD**	** $\frac{1}{\text{N}}|{\text{X}}_{\text{a}v\text{g}}\left(\text{f}\right){|}^{2}$ **
Power banks	$\frac{\sum_{f=n\hat{f}-f_{0}}^{n\hat{f}+f_{0}}|X_{channel}\left(f\right){|}^{2}}{\sum_{f=0}^{N}|X_{channel}\left(f\right){|}^{2}}$
Cosine correlation	$x_{channel}{\left(t\right)}^{*}\cos\left(2\pi n\hat{f}t\right)$

It is important to note that when completing the AttentionCARE protocol both the baseline phases and feedback phase are done in the same session, meaning that the classifier was trained immediately following the baseline phases on the data collected from that participant. This places an additional constraint on the model selected, as it is paramount that the training process is fast to ensure participants remain engaged. Thus, the best-performing classifier was chosen not just by the accuracy of the model but rather a combination of the accuracy and the time to train.

#### 2.5.2 Replication sample

Following the experiments with the preliminary sample to determine the best-performing classifier that meets all criteria previously outlined, the replication sample was used to evaluate the classifier in several ways. Firstly, an identical analysis, using five-fold cross validation was performed to establish if the model performance could be replicated on the replication sample based on a threshold determined by the preliminary sample. The threshold was chosen to be within 10% of the accuracy on the preliminary sample, the 10% tolerance is reasonable criteria as differences in the classifier performance across the two groups was expected since EEG signals vary across individuals (Schirrmeister et al., 2017; King et al., 2018). Due to the limited number of participants, a statistical analysis cannot be informative therefore the threshold was used to explore the replicability until more data is gathered. Additionally, the best-performing classifier on the preliminary sample was implemented into the protocol to calculate feedback during the feedback phase that the replication sample completed. The classifier was trained on an 80%−20% split of the baseline data to train and validate the model. Following training the model a posterior probability distribution was fit to the SVM to normalize the output between zero and one (Platt, 1999), thus the feedback score represents the probability that a participant is attending to the Gabor, relative to the face. The feedback given by the classifiers used during the feedback phase was validated on the baseline data to ensure that the probability scores were informative to participants. Additionally, an examination of the reliability and usability of the system was conducted on all phases of the AttentionCARE protocol completed by the replication sample to certify the stability of all phases in the protocol. Lastly, associations between participant performance during the feedback phase and their acceptability survey responses were examined.

## 3 Results

As described in the methodology, we utilized the preliminary sample to investigate several different types of SVM classifiers along with different feature types for implementation into the AttentionCARE protocol. After completing the protocol, the replication sample data was used to determine if the results from the preliminary sample were replicated, indicating a within-study replication. The validity of the feedback scores, or Gabor probabilities, were examined in the replication sample to ensure that the feedback given was significantly different when attending to the Gabor or Face. Furthermore, we tested whether the high internal reliability was preserved from our original work (Huang et al., 2022) after the SVM classifier was tested in an adolescent sample, which would represent a cross-study replication. Finally, exploratory analyses were conducted to assess the preliminary effectiveness of the feedback during the feedback phase.

### 3.1 Within-study implementation, replication, and validation of a participant-specific SVM classifier

#### 3.1.1 Implementation

The results of the participant-specific SVM experiments performed on the Preliminary Sample for each feature type are shown in Table 3. The table indicates which feature type was used along with the specific parameters, if applicable, for a given experiment. The parameters used are the number of harmonics, *n*, and the range of the filter banks, f_0_, and whether forward feature selection was used, FS.

**Table 3:** Average accuracy across the preliminary sample using power spectral density features (PSD), power bank features, and cosine correlation features (cosine corr) with different number of harmonics (*n*), frequency ranges (f_0_) and using the forward feature selection technique (FS).

**Feature type**	**Parameters**	**Linear**	**Polynomial**	**Radial basis function**
PSD	–	0.703 ± 0.204	0.700 ± 0.170	0.690 ± 0.208
PSD	FS	0.683 ± 0.198	0.683 ± 0.165	0.677 ± 0.212
Power bank	*n* = 1, f_0_ = 1.5	0.633 ± 0.172	0.607 ± 0.120	0.640 ± 0.103
Power bank	*n* = 2, f_0_ = 1.5	0.670 ± 0.135	0.703 ± 0.127	0.670 ± 0.157
Power bank	*n* = 3, f_0_ = 1.5	0.737 ± 0.107	0.757 ± 0.102	0.710 ± 0.152
Power bank	*n* = 3, f_0_ = 1.5, FS	0.800 ± 0.096	0.803 ± 0.119	0.800 ± 0.102
Power bank	*n* = 1, f_0_ = 2	0.717 ± 0.120	0.690 ± 0.148	0.620 ± 0.145
Power bank	*n* = 2, f_0_ = 2	0.743 ± 0.89	0.663 ± 0.157	0.703 ± 0.112
Power bank	*n* = 3, f_0_ = 2	0.770 ± 0.093^*^	0.730 ± 0.126	0.757 ± 0.138
Power bank	*n* = 3, f_0_ = 2, FS	0.817 ± 0.86	0.773 ± 0.124	0.857 ± 0.082
Cosine Corr	*n* = 1	0.503 ± 0.114	0.540 ± 0.064	0.560 ± 0.134
Cosine Corr	*n* = 2	0.527 ± 0.058	0.553 ± 0.049	0.580 ± 0.134
Cosine Corr	*n* = 3	0.560 ± 0.069	0.613 ± 0.066	0.617 ± 0.132
Cosine Corr	*n* = 3, FS	0.697 ± 0.056	0.670 ± 0.115	0.750 ± 0.099

The performance of the classifier selected for further testing in the replication sample is highlighted and marked with an asterisk.

As seen in the table, the best-performing classifier, based on the average accuracy across the preliminary sample, was a linear SVM with power bank features using a frequency range of f_0_ = 2, *n* = 3 harmonics, and the forward feature selection technique. However, our participant-specific SVM experiments also revealed that training this classifier required an increased time to complete (5–10 min), given the extended time requirements imposed by the forward feature selection method. As described in Section 3.2, there is an additional constraint on time to train the model, which when using the forward feature selection technique cannot be met. Past research has demonstrated that shorter ABMT protocols are associated with more robust clinical effects (Price et al., 2017), thus we chose to minimize any unnecessary delays in the current protocol. Due to this, the best classifier not using forward feature selection was chosen for the protocol, which was a linear SVM with power bank features using a frequency range of f_0_ = 2, *n* = 3 harmonics. The average accuracy of this classifier was 0.770 ± 0.093, establishing a replication threshold of 0.693 – 0.847.

#### 3.1.2 Replication

In the replication sample, the linear SVM with power bank features using a frequency range of f_0_ = 2, *n* = 3 harmonics was found to have an average accuracy of 0.710 ± 0.115, which is within the threshold range considered indicating a replication of the findings from the preliminary sample.

#### 3.1.3 Validation

To validate the performance of the linear SVM with power bank features using a frequency range of f_0_ = 2, *n* = 3 harmonics used in the replication sample, we conducted analyses to determine if the classifier could discriminate between SSVEP responses for a participant attending to either the Gabor or the face. When participants were instructed to pay attention to the Gabor and ignore the face in Baseline 1, the probability scores were significantly higher (*M* = 0.591, *SD* = 0.214) compared to when they were asked to focus on the face and ignore the Gabor in Baseline 2 (*M* = 0.286, *SD* = 0.183), *t*(4)=3.885, *p* = 0.018, Cohen's *d* = 1.737. This result indicates that the classifier trained for the AttentionCARE protocol successfully discriminated attentional competition between competing visual stimuli that overlap in time and space.

### 3.2 Cross-study replication of the internal consistency of the AttentionCARE protocol

To replicate the findings from our previous work regarding internal consistency of the AttentionCARE Protocol (Huang et al., 2022), we performed an identical analysis on the split-half reliability during all phases completed by the replication sample. We found excellent even-odd split-half reliability (Guttman coefficients ranging from 0.963 to 0.998), demonstrating that the reliability of the protocol was preserved in an adolescent sample and while using a participant-specific SVM classifier. Of note, a Guttman coefficient closer to 1 indicates higher internal consistency of the calculated probability scores. Internal reliability of a protocol is important to consider as feedback with low reliability may not be useful to a participant since the effectiveness of the techniques learned to redirect their attention would not be reflected in unreliable feedback.

### 3.3 Evaluation of feedback effects observed during the AttentionCARE protocol

Table 4 shows the average Gabor probabilities classified during the feedback phase, split by participant ID. The table also presents average Gabor probabilities across the number of times a face was seen and in the first vs. second half of the feedback phase. Of note, probability scores >0.50 indicated that the Gabor “won” the competition for attention, as determined by the classifier. If the feedback protocol was working as expected, we would expect that most participants would show Gabor probabilities >0.50. In contrast, as shown in Table 4, only two out of five participants attended more to the Gabor, relative to the face, on average during the feedback phase.

**Table 4:** Average Gabor probability scores observed across the feedback phase, split by participant.

**Participant**	**Average**	**Number of times a face is seen**					**Half of feedback phase**	
		**1**	**2**	**3**	**4**	**5**	**First**	**Second**
1	0.70	0.67	0.74	0.71	0.69	0.68	0.67	0.73
2	0.25	0.27	0.29	0.21	0.27	0.22	0.32	0.18
3	0.68	0.70	0.72	0.63	0.68	0.69	0.74	0.62
4	0.20	0.18	0.20	0.17	0.25	0.18	0.18	0.21
5	0.45	0.42	0.43	0.46	0.44	0.50	0.53	0.37

Average Gabor probabilities are also compared across the number of times a face was seen and in the first vs. second half of the feedback phase.

To probe this unexpected finding, we conducted exploratory analyses to consider whether Gabor probabilities would increase as the number of times each face was seen, as this would indicate that the participant was learning from feedback given during previous trials. In addition, comparisons of the first vs. the second half of the feedback phase could be used to describe differences in performance after just being exposed to the feedback phase compared to when a participant has more experience. To statistically examine changes in Gabor probability scores by the number of times a face was shown (i.e., trial 1 through 5) and over time (first half vs. second half of the feedback phase), we conducted a 2 (Time: first half, second half) × 5 (Trial: 1–5) repeated measures ANOVA with Gabor probabilities serving as the dependent variable. The main effects of Time, *F*_(1,4)_ = 2.135, *p* = 0.218, $\eta_{\text{p}}^{2}$ = 0.348, and Trial were nonsignificant, *F*_(4,4)_ = 1.412, *p* = 0.275, $\eta_{\text{p}}^{2}$ = 0.261, indicating that the Gabor probabilities did not differ significantly across time or trials. In addition, the Time × Trial interaction was nonsignificant, *F*_(4,4)_ = 0.543, *p* = 0.707, $\eta_{\text{p}}^{2}$ = 0.120.

Finally, to consider whether there were individual differences associated with participant performance, we examined correlations between participants' average Gabor probabilities during the feedback phase and responses from the acceptability questionnaire. These are depicted in Figure 3. Gabor probability scores were positively correlated with perceived fairness, efficacy, clarity, and value of the protocol, as well as negatively correlated with effort.

## 4 Discussion

Affect-biased attention is a well-established cognitive vulnerability implicated in the development and maintenance of depression that is known to emerge during adolescence (Gibb et al., 2023). The current study describes the development and implementation of the novel BCI, AttentionCARE, designed to modify affect-biased attention in a sample of adolescents enriched for risk for future depression (i.e., adolescent girls, 70% of whom also had a history of maternal MDD during their lifetime). To address the lack of reproducibility and reliability in past BCI applications, our study hypotheses emphasized findings that we could show to be replicable and verifiable. Specifically, we conducted a successful replication of our earlier pilot findings in adults (Huang et al., 2022), which showed that discriminant SSVEP responses could be generated by our BCI with high levels of internal reliability. However, in the current study, we achieved these results by developing a novel participant-specific SVM to calculate the probability that a participant was exhibiting high levels of goal-oriented attention (i.e., attention to the Gabor in the AttentionCARE protocol) compared to stimulus-driven attention (i.e., attention to distracting angry and sad faces) and testing it in a sample of high-risk adolescents. High internal consistency that can be reproduced across developmental stages (adults vs. adolescents) and feedback classifiers (generalized vs. person-specific) signifies that our BCI can be used to deliver reliable feedback to participants, which is an essential prerequisite of learning to modify affect-biased attention. Regarding within-study replication, our hypothesis that the performance of our novel SVM would be equivalent in both our preliminary and replication samples was confirmed. Furthermore, the probability scores generated by the participant-specific SVM were validated by our findings that there is a significant difference in scores when attending to the Gabor vs. the face. Together, these results indicate that performance, as indicated by our classifier, is generalizable across a replication sample, and not specific to the performance of the participants in the preliminary sample. All of these factors are critical when designing reliable and reproducible BCI applications.

Our findings suggest that further testing of our BCI to modify affect-biased attention is warranted. The participant-specific SVM used to generate probability scores demonstrated acceptable accuracy (71%−77%), suggesting that it can be used to provide personalized feedback to participants. This is in line with the theoretical promise of using EEG-based neurofeedback to modify affect-biased attention, as EEG offers the marriage of precise neural measures and feasible clinical translation (Woody and Price, 2022). In addition, the current findings suggest that our BCI overcomes some of the reliability and interpretability challenges associated with previous ABMTs (Price et al., 2015; Rodebaugh et al., 2016; Xia et al., 2023). Finally, our use of AR technology is a novel application that could be used to enhance participant comfort and engagement.

**Figure 5:**
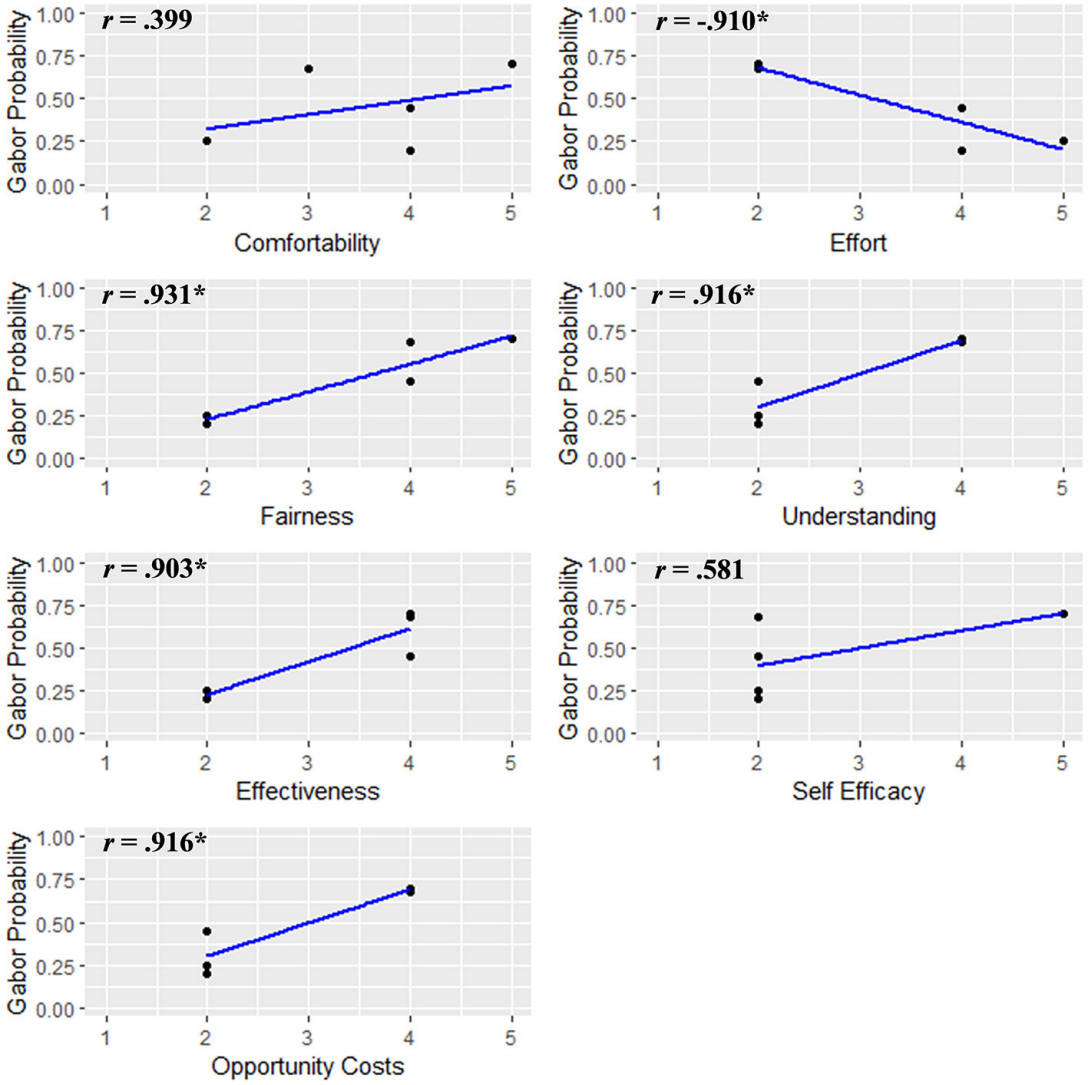
Scatter plots for each acceptability survey question and the accompanying average Gabor probability during the feedback phase. *p < 0.05.

Notably, our novel SVM classifier revealed that the majority of participants completing the feedback phase did not demonstrate the expected improvement in performance over time. Although no clear evidence of learning effects were observed, we hypothesize that participants may need to complete the AttentionCARE protocol several times to effectively learn to direct their affect-biased attention, but a larger sample size is needed before inferences can be drawn about learning effects and how to improve them. In spite of the fact that there was no demonstrated improvement during the feedback phase, the validation of the BCI's reliability and reproducibility is an important first step into clinical efficacy. Now that this has been demonstrated future studies can focus on exploring therapeutic applications of the BCI to establish dosing and efficacy guidelines and develop precision medicine protocols that use personalized BCIs to target specific patterns of affect-biased attention. Additionally, our exploratory analyses revealed several patterns that may highlight avenues for future research. We observed large and significant correlations between average Gabor probability scores and responses on our acceptability questionnaire, see Figure 5, such that probability scores were positively correlated with participants' perceptions of fairness, efficacy, clarity, and the value of the protocol and negatively correlated with the perceived effort required to complete it. The feedback from individuals identified as having lower performance on the protocol suggests that they might not have actively participated due to potential factors such as lack of comprehension, interest, or motivation, these findings pend replication in larger samples. To better ensure clinically meaningful effects the future sample sizes should be powered to find medium or larger effects.

In addition to its strengths, our study also had several limitations. The current analyses were restricted to 10 adolescents, and only five adolescents completed the feedback phase. Future studies with larger sample sizes are needed to test learning effects during the feedback phase. Second, although we have demonstrated that AR can be used to direct participants' attention, we have not yet realized the full potential of AR in the AttentionCARE protocol. In the current protocol, negative distractors consist of static images of adolescent actresses displaying sad and angry facial expressions. However, AR has the potential to use person-specific images, such as images from a user's cell phone, in immersive mixed-reality environments, which could help improve participant engagement and increase ecological validity. Finally, we have not yet tested the AttentionCARE BCI in a sample of adolescent boys, which may limit the generalizability of our results to only the highest risk adolescents (i.e., daughters of depressed mothers; Goodman, 2007). Future studies will be needed to test our protocol in a fully representative sample of adolescents and to determine if there are sex differences in performance during the protocol. Based on prior research we wouldn't expect to see sex differences in affect-biased attention (e.g., Gibb et al., 2023), but future studies will benefit from examination of potential sex differences in dosing and clinical effects.

In conclusion, our study describes an advancement in the development and implementation of a novel BCI to modify affect-biased attention in adolescents at high risk for depression. The successful within- and between-study replications underscores the robustness and reliability of our AttentionCARE BCI, for use with both adolescents and adults. The demonstrated accuracy of our participant-specific SVM shows promise for the delivery of personalized feedback in future ABMTs. Future studies exploring dosing and efficacy in larger samples, with more diverse participant demographics, and refined AR applications are essential for a more comprehensive understanding of the protocol's potential for modifying affect-biased attention, which would inform future clinical trials.

## Data availability statement

The raw data supporting the conclusions of this article will be made available by the authors upon request.

## Ethics statement

The studies involving humans were approved by University of Pittsburgh IRB. The studies were conducted in accordance with the local legislation and institutional requirements. Written informed consent for participation in this study was provided by the participants' legal guardians/next of kin.

## Author contributions

RG: Conceptualization, Data curation, Formal analysis, Methodology, Software, Validation, Writing – original draft, Writing – review & editing. NM: Data curation, Investigation, Writing – original draft, Writing – review & editing. XH: Data curation, Software, Writing – review & editing. AW: Data curation, Investigation, Writing – review & editing. RP: Conceptualization, Investigation, Supervision, Writing – review & editing. SO: Conceptualization, Funding acquisition, Investigation, Resources, Writing – review & editing. MA: Conceptualization, Funding acquisition, Methodology, Resources, Supervision, Writing – review & editing. MW: Conceptualization, Funding acquisition, Investigation, Methodology, Resources, Supervision, Writing – original draft, Writing – review & editing.
